# Hydrogen‐rich water and caffeine for alertness and brain metabolism in sleep‐deprived habitual coffee drinkers

**DOI:** 10.1002/fsn3.2480

**Published:** 2021-07-19

**Authors:** Nikola Todorovic, Dragana Zanini, Valdemar Stajer, Darinka Korovljev, Jelena Ostojic, Sergej M. Ostojic

**Affiliations:** ^1^ Applied Bioenergetics Lab Faculty of Sport and PE University of Novi Sad Novi Sad Serbia; ^2^ Faculty of Health Sciences University of Pecs Pecs Hungary

**Keywords:** alertness, brain metabolism, choline‐to‐creatine ratio, hydrogen, sleep

## Abstract

The main aim of this randomized‐controlled cross‐over interventional trial was to assess the acute effects of taking a single dose of hydrogen‐rich water (HRW), and compare it with caffeine, HRW plus caffeine, and control water, for alertness, brain metabolism, brain and oxygen saturation, and self‐reported adverse events in healthy men and women who were habitual coffee drinkers and were sleep‐deprived for 24 hr. Sixteen apparently healthy young adults (8 men and 8 women; age 24.0 ± 3.5 years) were allocated in a cross‐over design to receive a single‐dose drink of HRW (8 ppm), caffeine (50 mg), HRW plus caffeine, or control drink (tap water) in the morning after 24‐hr sleep deprivation and 12‐hr fasting. The primary and secondary outcomes were assessed at baseline (pre‐intervention) and 15‐min follow‐up. Significantly less time was needed to complete trail‐making test after both HRW and HRW plus caffeine compared with the control drink (*p* < .05). The number of errors in the symbol digit modalities test was significantly lower after drinking HRW or caffeine than control drink (*p* < .05). Both HRW and caffeine significantly increased the choline‐to‐creatine ratio in several brain regions (frontal white and gray matter), while HRW and the combination intervention also affected brain metabolism in the paracentral brain. No participants reported any side effects from any intervention. The attention enhancement driven by HRW appears along with changes in brain metabolism. Being generally recognized as a safe intervention, hydrogen could be thus recommended as a novel intervention that upholds attention in stressed conditions, with its metabolic footprint likely different from caffeine.

## Graphical abstract

Drinking a single dose of hydrogen‐rich water (HRW) improves alertness in sleep‐deprived young men and women. The attention enhancement driven by HRW appears to go with notable changes in brain metabolism, illustrated by higher choline‐to‐creatine ratio levels in the frontal and paracentral brain. Being a harmless intervention, HRW could be thus recommended as a novel food intervention that upholds attention in stressed conditions, with its metabolic footprint likely different from caffeine.

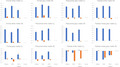

## 1 INTRODUCTION

Molecular hydrogen (dihydrogen, H_2_) is a novel biotherapeutic gas that appears effective in various health conditions. Hydrogen‐rich water (HRW: also known as hydrogen‐infused water) emerges as the most common vehicle to deliver dihydrogen in biomedicine, and over 1,500 studies published during the past decade or so confirm the favorable effects of drinking HRW in both animal and human trials (for a detailed review see Yang et al., 2020). Due to its pleiotropic biological activity and ability to easily cross the blood‐brain barrier (Ohta 2012), HRW was found to ameliorate patient‐reported scores in Parkinson's disease (Yoritaka et al., 2013), increase mood, anxiety, and autonomic nerve function in daily life (Mizuno et al., 2018) and reduce the presence and severity of symptoms of mild traumatic brain injury (Javorac et al. 2019). The beneficial effects of dihydrogen in neurology are attributed to its role in neurometabolic processes, including bioenergetics, anti‐oxygenation, anti‐inflammation, and anti‐apoptosis, (Iketani & Ohsawa, 2017; Ostojic, 2017). However, no human studies so far have evaluated the immediate impact of dihydrogen on brain metabolism. We recently found that HRW is superior to caffeine, a well‐known cognitive enhancer, to improve orientation‐specific alertness in healthy men and women (Zanini et al., 2020). A limited number of brain‐specific outcomes and biomarkers used in this pilot trial require additional investigation to compare HRW and caffeine for brain function. Therefore, the main aim of the present study was to evaluate the acute effects of drinking single‐dose HRW, and compare it with caffeine, HRW plus caffeine, and control water (no dihydrogen and caffeine) for alertness, brain metabolism, brain and oxygen saturation, and self‐reported adverse events in healthy men and women who were sleep‐deprived for 24 hr.

## 2 METHODS

### 2.1 Participants

Sixteen healthy adults (8 men and 8 women; age 24.0 ± 3.5 years, body mass index 23.5 ± 2.4 cm) signed an informed consent to voluntarily participate in this randomized‐controlled cross‐over interventional trial. Inclusion criteria were as follows: age 18 to 30 years, healthy body mass index (*e.g*., 18.5 to 25.0 kg/m^2^), no current chronic diseases or acute disorders, habitual coffee drinking, and sleep deprivation of 24 hr. Exclusion criteria included a previous history of dietary supplement use during the four weeks before the study commences and caffeine intake 12 hr before the trial. The minimal sample size (*n* = 16) was calculated using power analysis (G*Power 3.1), with the effect's size set at 0.50 (medium effect), alpha error probability 0.05, and power 0.80 for four groups, and eight measurements of study outcomes. The primary outcome was the change in reaction time for the attention network test (see below) at baseline and 15‐min postadministration. The study design was approved by the local IRB at the University of Novi Sad (# 2‐CFHRW/2020), with the study systematized following the Declaration of Helsinki and International Conference of Harmonization Efficacy Guidelines E6.

### 2.2 Experimental interventions

**FIGURE 1:**
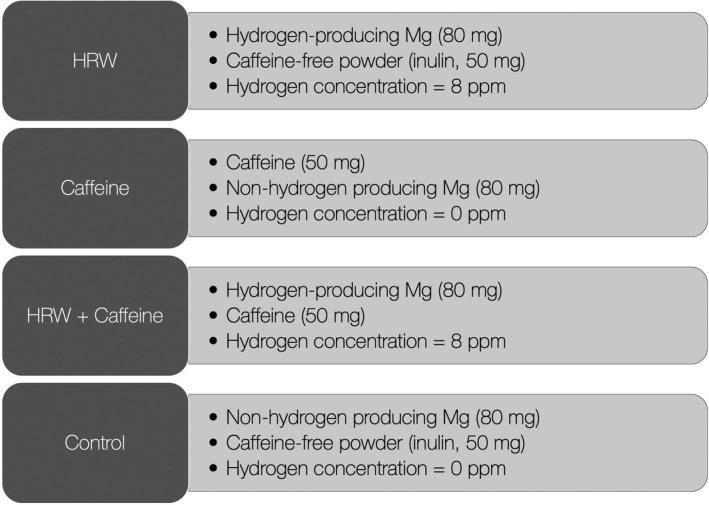
The composition of experimental interventions. HRW—ahydrogen‐rich water

All volunteers were allocated in a cross‐over design to receive a single‐dose drink of HRW, caffeine, HRW plus caffeine, or control drink in the morning after 24‐hr sleep deprivation and 12‐hr fasting. The composition of each drink is presented in Figure 1, with all experimental interventions produced by dissolving specific tablets/powders into a cup of lukewarm water (500 ml). HRW and nonhydrogen producing magnesium tablets were provided by HRW Natural Health Products Inc. (New Westminster, BC, Canada), and caffeine anhydrous powder was purchased by Proteos (Zagreb, Croatia). Dihydrogen in HRW was produced by the following reaction: Mg + H_2_O → H_2_ + Mg(OH)_2_, with magnesium used in HRW was elemental magnesium. The content of dihydrogen in all experimental drinks was measured by gas chromatography, as previously described (Zanini et al., 2020). All drinks were similar in appearance, texture, and sensory characteristics, and normalized for total magnesium amount. A wash‐out period of 7 days was set to prevent the residual effects of interventions across study periods.

**FIGURE 2:**
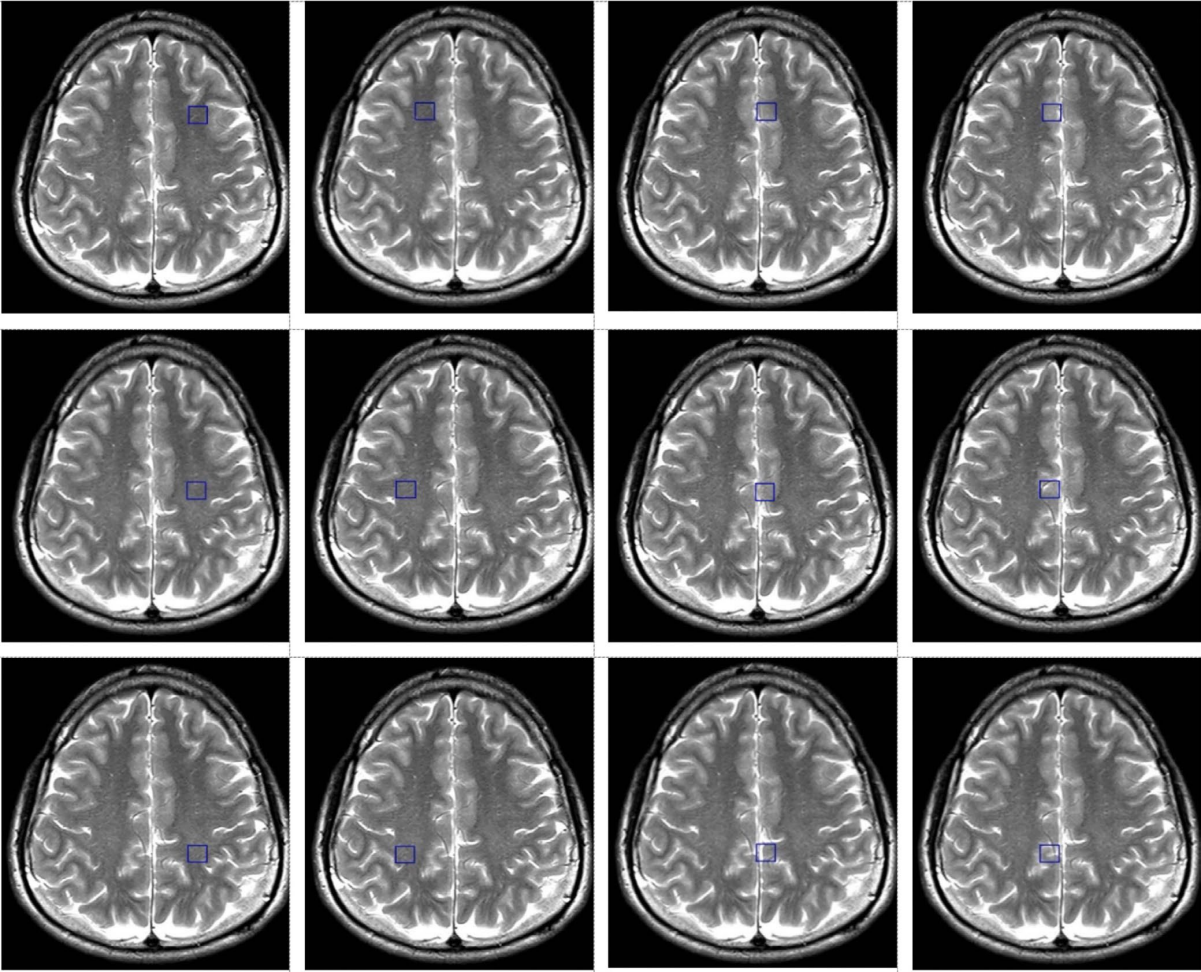
Location of the individual voxels (blue square) evaluated during the study

Sleep deprivation was secured by keeping participants awake in a sleep quarantine room within FSPE Applied Bioenergetics Lab under continuous control of research staff for 24 hr before the experimental drinks’ intervention. Outcomes assessed at baseline (pre‐intervention) and 15‐min follow‐up were visual analogue scale (VAS) for alertness (Srivastava et al., 2013), and the attention network test (ANT) with subscales for alerting, orienting, and executive control (Macleod et al., 2010). Trail‐making test A was also employed as an indicator of visual scanning, graphomotor speed, and executive function (Llinàs‐Reglà et al., 2017), along with symbol digit modalities test (SDMT) (Smith, 2000). Brain oxygenation (SbO_2_) and hemoglobin index (tHb) in the prefrontal cortex was monitored with 4‐optode (680–800 nm) functional near‐infrared spectroscopy sensor (Fortiori Design LLC, Hutchinson, Minnesota), while peripheral capillary oxygen saturation (SpO_2_) was monitored with fingertip oximeter (Barrington Diagnostics, Barrington, IN). Magnetic resonance imaging and spectroscopy were performed on a 1.5 T Siemens Avanto scanner (Erlangen, Germany), using matrix head coil (receiver coil) in circularly polarized mode as previously described (Ostojic et al., 2017). Water‐suppressed proton 2D Spectroscopic Imaging (CSI) and single‐voxel data sets were acquired with point‐resolved spectroscopy TR/TE of 1500/135 ms. The first CSI slab [field of view (FOV) 160 × 160 × 160 mm; voxel of interest (VOI) 80 × 80 × 80 mm, thickness 10 mm] was positioned parallel to the axial images, immediately above the corpus callosum along the anterior–posterior commissure, to encompass the semioval white matter and the cortical gray matter. The number of phase‐encoding steps (scan resolution) was 12 in all directions (R‐L, A‐P, and F‐H). The number of reconstructed spectra (interpolation resolution) was 12 in all directions, resulting in a VOI of 10 × 10 × 10 mm. Twelve phase‐encoding steps and reconstructed spectra were recorded, resulting in a VOI of 8 × 8 × 15 mm. The number of acquisitions was 4, with a scan time of 7 min 12 s for each CSI acquisition. A nonwater‐suppressed CSI and SVS data were also obtained with the same geometrical parameters (e.g., one average for CSI, sixty‐four averages for SVS) to provide an internal water reference for the absolute quantification of relevant metabolic ratios. The weighted phase‐encoding scheme was applied. Interfering signal contributions from areas outside the VOI were suppressed by six saturation regions, manually positioned along each VOI margin. The homogeneity of the magnetic field was optimized using manual shimming. Special care has been taken to position each region of interest in the exact location for all subjects to achieve the highest possible level of reproducibility. Spectra were evaluated from 12 individual voxels: six areas were located in bilateral parasagittal anterior, middle, and posterior cortices, characterized primarily by frontal, paracentral, and parietal mesial gray matter, and six were in lateral anterior, middle, and posterior regions containing predominantly frontal, precentral, and parietal white matter (Figure 2). A total of 285 spectra were analyzed in this study. The CSI raw data were evaluated using a commercially available spectral analysis software package (Syngo Multi‐Modality Workplace, version VE23A, Siemens, Erlangen, Germany). The postprocessing protocol included water reference processing by averaging 20 adjacent points, removing the residual water signal from the spectrum by subtracting it from the time signal and frequency shift correction of the water signal, Hanning filter 512 ms width, Zero filling from 512 to 1,024 data points, and Fourier transformation. Signals for creatine (3.04 ppm), choline (3.2 ppm), and NAA (2.02 ppm) were quantified using Gaussian curve fittings. The choline‐to‐creatine and choline‐to‐*N*‐acetyl aspartate (NAA) ratios were calculated from areas under the respective signals. The area of water signal for each processed voxel was assessed from the scan without water suppression. The quantification of the SVS data was performed offline in the time domain using the jMRUI software package. For metabolite spectra processing, the remaining water signal was removed using an HLSVD filter, and amplitudes of choline, creatine, and NAA signals were calculated with the AMARES method (Vanhamme et al. 1997). The amplitude of the water signal for each processed voxel was assessed from the scan without water suppression. Mono‐exponential spin‐lattice and spin‐spin relaxation were assumed, and published values of T1 and T2 relaxation times of water and respective metabolites measured at 1.5 T in the gray and white matter of healthy volunteers were used for relaxation corrections (Bajzik et al. 2008). The volunteers were also instructed to report any side effects of each intervention (*e.g*., palpitations, gut disturbances, and headache) throughout the study with an open‐ended questionnaire.

### 2.3 Statistical analyses

Friedman test was used to establish whether significant differences existed between different experimental drinks over time of intervention (baseline versus. 15‐min postadministration), with post hoc test used to identify differences between individual sample pairs. Data were analyzed using SPSS Statistics for Mac Version 24.0 (IBM, Armonk, NY), with the significance level set at *p* <.05.

## 3 RESULTS

All volunteers completed all four trials, with no participants reported any side effect of each intervention. Changes in alertness biomarkers were depicted in Table 1. A significant treatment versus. time interaction was found for reaction time, trail‐making test duration, and SDMT outcomes (*p* <.05). In particular, a mean change in reaction time at 15‐min follow‐up was different between caffeine and HRW (*p* <.05), and caffeine and HRW plus caffeine (*p* <.05). A significantly less time was needed to complete trail‐making test after both HRW and HRW plus caffeine compared with the control drink (*p* <.05). Finally, the number of errors in the SDMT test was significantly lower after drinking HRW or caffeine than control drink (*p* <.05). No differences were found for SbO2, tHb, and SpO2 during the trial (*p* >.05).

**TABLE 1:** Changes in alertness and oxygenation outcomes during the study

	Baseline	At 15‐min follow‐up				P *	Post hoc
		Control	Caffeine	HRW	HRW +Caffeine		
VAS alertness (score)	4.5 ± 2.6	5.3 ± 2.2	5.0 ± 2.4	5.6 ± 2.3	6.5 ± 1.9	0.095	‐
ANT alerting (ms)	37.0 ± 26.7	45.6 ± 32.4	43.6 ± 30.3	35.3 ± 37.1	40.3 ± 32.7	0.802	‐
ANT orienting (ms)	37.6 ± 27.2	46.1 ± 28.5	46.3 ± 22.5	42.6 ± 25.9	46.8 ± 25.6	0.963	‐
ANT executive control (ms)	143.2 ± 57.7	153.3 ± 62.7	127.4 ± 35.1	136.1 ± 88.6	107.3 ± 42.2	0.070	‐
Reaction time (ms)	567.3 ± 81.2	586.9 ± 76.5	578.7 ± 71.7	609.4 ± 68.7	626.9 ± 90.4	0.041	^d e^
Test accuracy (%)	96.6 ± 3.4	94.4 ± 9.9	98.6 ± 1.3	97.9 ± 3.1	98.6 ± 1.4	0.072	‐
Trail‐making test A (ms)	21.6 ± 5.0	22.8 ± 5.1	19.3 ± 7.3	16.6 ± 4.9	17.0 ± 3.7	0.001	^b c^
SDMT (number of symbols)	53.2 ± 7.3	58.6 ± 6.1	61.2 ± 8.7	62.9 ± 9.7	59.1 ± 10.5	0.045	^a b^
SDMT (errors)	1.1 ± 2.5	1.3 ± 2.0	0.6 ± 0.7	0.7 ± 0.9	1.1 ± 0.9	0.297	‐
Brain oxygenation (%)	67.5 ± 6.0	62.4 ± 6.6	63.7 ± 9.0	63.1 ± 8.1	60.7 ± 5.8	0.592	‐
Total hemoglobin (umol/L)	13.0 ± 0.1	12.9 ± 0.2	13.0 ± 0.2	13.0 ± 0.2	12.9 ± 0.3	0.803	‐
Peripheral oxygen saturation (%)	98.5 ± 0.7	98.5 ± 0.5	98.1 ± 1.4	98.3 ± 1.2	98.7 ± 0.5	0.899	‐

Note

Values are mean ± *SD*.

Abbreviations: ANT, attention network test;^b^, control versus. HRW; ^c^, mcontrol versus. HRW plus caffeine; ^d^, caffeine versus. HRW; ^e^, caffeine versus. HRW plus caffeine;HRW, hydrogen‐rich water. * *P* value from two‐way mixed ANOVA (treatment versus. time interaction). Post hoc superscript indicates a significant difference at *p* <.05 for the following sample pairs: ^a^, control versus. caffeine; SDMT, symbol digit modalities test; VAS, visual analogue scale.

**FIGURE 3:**
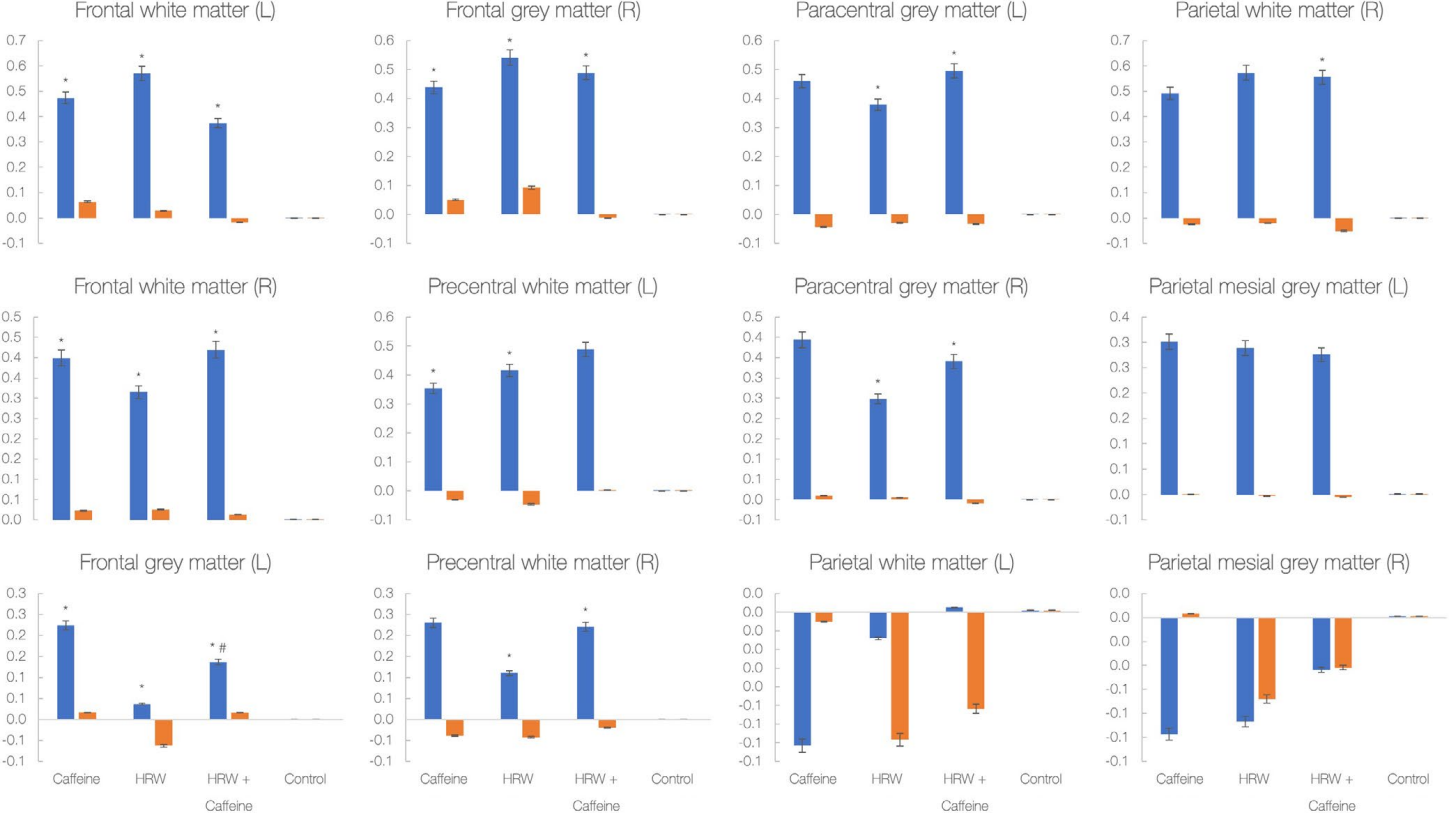
Changes in the choline‐to‐creatine ratio (blue) and choline‐to‐N‐acetyl aspartate (NAA) ratio (orange) in 12 various brain regions during the study. Y‐axis labels absolute values for metabolic ratios; each bar indicates changes from baseline to 15‐min follow‐up. Asterisk (*) indicates a significant difference at *p* <.05 between the experimental drink and control drink; # indicates a significant difference at *p* <.05 between hydrogen‐rich water (HRW) and HRW plus caffeine

Figure 3 depicts changes in brain metabolic ratios across twelve different brain locations. It appears that a significant treatment versus. time interaction was found for the changes in the choline‐to‐creatine ratio in 9 out of 12 regions (*p* <.05). Caffeine significantly increased the choline‐to‐creatine ratio comparing to control drink in 5 locations (*p* <.05), HRW significantly increased the ratio in 8 locations (*p* <.05), and HRW plus caffeine significantly increased the ratio in 8 locations (*p* <.05). Besides, a combination was superior to HRW to increase the choline‐to‐creatine ratio in left frontal gray matter (*p* <.05). No differences were found between interventions for the choline‐to‐NAA ratio across the trial (*p* >.05).

## 4 DISCUSSION

The present study corroborated preliminary findings that hydrogen‐rich water could acutely affect biomarkers of alertness in sleep‐deprived men and women. Specifically, HRW was superior to control drink to improve trail‐making and SDMT performance, while caffeine outperformed HRW for reaction time. The effects seen here were accompanied by changes in brain metabolism. Both HRW and caffeine significantly increased the choline‐to‐creatine ratio in several brain regions (*e.g*., frontal white and gray matter), while HRW and the combination intervention also affected brain metabolism in the paracentral brain. These findings suggest that HRW and caffeine probably affect different domains of alertness and stimulate metabolism in separate brain segments when administered acutely in sleep‐deprived volunteers.

During the past decade or so, molecular hydrogen has emerged as an innovative agent in human neuroscience and clinical neurology. The effects of this simple biomedical gas on brain performance have been evaluated in a handful of interventional trials, spanning from brain trauma to neurodegenerative and cerebrovascular diseases. For instance, hydrogen improved the brain MRI indices in the acute brain stem infarct sites (Ono et al., 2011), positively affected patient‐reported scores in Parkinson's disease (Yoritaka et al., 2013), attenuated the stroke severity in patients with cerebral infarction (Ono et al., 2017), increased mood, anxiety, and autonomic nerve function in healthy volunteers (Mizuno et al., 2018), reduced the presence and severity of symptoms of mild traumatic brain injury (Javorac et al. 2019), and improved cognitive function in community‐dwelling older women (Korovljev et al., 2020). Although those studies showed promising results of medium‐ to long‐term intervention with hydrogen on various indices of brain function, few trials endeavored to evaluate short‐term effects of hydrogen on brain performance. Besides, no neuroimaging studies so far evaluated the immediate effects of hydrogen on brain metabolism in separate brain areas involved in perception, executive function, and connectivity, or contrast hydrogen with other brain‐stimulating agents. Our group's previous pilot investigation was arguably the first interventional study that compared the acute effects of single‐dose HRW and caffeine on brain performance among healthy men and women who were sleep‐deprived for 24 hr (Zanini et al., 2020). Both interventions acutely affected markers of alertness, yet caffeine induced a drop in alerting and executive control at 15‐min follow‐up, while HRW caused a reduction in the orientation at postadministration. Although no differences were found between interventions for all evaluated outcomes of alertness, the authors suggested that HRW and caffeine might have impacted different alertness domains, with dihydrogen improving orienting to sensory stimulation. In contrast, caffeine alters awareness and executive attention. The current study findings extend previous research by showing stimulating effects of acute dihydrogen and caffeine intake on brain performance and metabolism in stressed individuals, with HRW having appeared to tackle different brain regions compared with caffeine. We reported no significant differences in alertness outcomes among interventions, yet subjectively reported scores for alertness, executive control, and test accuracy strongly tended to be improved by all three experimental interventions at follow‐up, with HRW plus caffeine being the most prominent. Despite that, HRW and HRW plus caffeine were superior to control drink to complete trail‐making tests in a shorter time, suggesting improved visual attention and task switching for both interventions. In addition, both caffeine and HRW were better than control drink to complete SDMT, perhaps due to intervention‐driven stimulation of attention, perceptual speed, motor speed, and visual scanning reported previously (Zanini et al., 2020). Interestingly, reaction time was different for caffeine versus. both HRW and HRW plus caffeine, with all interventions provoked a delay in reaction time at 15‐min follow‐up. To our knowledge, this is the first human study that evaluated the acute effects of dihydrogen on brain metabolism. We found that the ameliorated brain function induced by HRW and caffeine appears to be accompanied by brain tissue metabolism adjustments. For instance, caffeine and HRW increased the choline‐to‐creatine ratio, an indicator of brain viability, in frontal bilateral white and gray matter, while HRW (and HRW plus caffeine) additionally elevated the ratio in the paracentral brain. This suggests that both interventions may positively affect region‐specific brain metabolism linked to executive functions, attention, and problem‐solving (frontal and prefrontal lobules), while HRW perhaps can also target brain regions relevant for orienting (paracentral lobule). A correlation between cognition and region‐specific choline/creatine ratio has been confirmed previously (Ben Salem et al., 2008), with trail‐making test results appears to be relevant not only for the frontal areas but also for more remote areas such as the thalamus, the insula, and the deep periventricular white matter. Also, we found that the combination of HRW and caffeine was equivalent or better than individual components for several alertness outcomes and brain metabolism indices. This perhaps suggests that HRW and caffeine could be consumed together while each component affects various domains of attention and brain metabolism.

Caffeine withdrawal should also be considered for study findings interpretation, with people experiencing caffeine withdrawal often experience symptoms such as headache, dizziness, fatigue, brain fog, and negative mood (Heatherley, 2011). The control group and HRW group could possibly be experiencing caffeine withdrawal symptoms while HRW might have some effect on caffeine withdrawal symptoms; future studies should recruit experimental subjects who do not habitually drink or eat caffeine or similar substances, such as theophylline. In addition, caffeine acts primarily on adenosine receptors (Costenla et al., 2010), which may cause nerve cells to speed up, since adenosine primarily causes drowsiness, and the caffeine blocks adenosine from the receptors, so the nerve cells tend to speed up and causes an alerting response. HRW might also interact with adenosine receptors, and this interaction should be investigated in future research. Finally, a recent study indicated that regular caffeine intake can cause minor gray matter atrophy, although it is reversible with caffeine cessation (Lin et al., 2021); this requires monitoring of structural brain changes in subsequent interventional studies with caffeine and caffeine‐free beverages.

The study strengths include the use of randomized‐controlled cross‐over design for administering HRW, and the relatively robust methodology to evaluate both participant‐ and clinician‐reported brain function outcomes. Our study is limited by containing a sample of young healthy subjects, so exploration on the effects of acute HRW administration in older adults or clinical populations with brain diseases is needed in order to ascertain benefits for these populations. Further studies should enroll these critical groups and perhaps evaluate the role of prolonged dihydrogen intake in experimental and clinical neurology.

## 5 CONCLUSION

Drinking a single dose of hydrogen‐rich water improves trail‐making test performance and reduces the number of errors in symbol digit modalities test in coffee habituated sleep‐deprived young adults. The attention enhancement driven by HRW appears to go with notable changes in brain metabolism, illustrated by higher choline‐to‐creatine ratio levels in the frontal and paracentral brain. Being generally recognized as safe intervention (Food and Drug Administration., 2014), hydrogen could be thus recommended as a novel intervention that upholds attention in stressed conditions, with its metabolic footprint likely different from caffeine.

## CONFLICTS OF INTEREST

The authors declare no conflicts of interest.

## ETHICAL APPROVAL

The study was approved by the local IRB at the University of Novi Sad (# 2‐CFHRW/2020), with the study systematized following the Declaration of Helsinki and International Conference of Harmonization Efficacy Guidelines E6.

## Data Availability

The data that support the findings of this study have already been included in the manuscript. Raw data are available from the corresponding author upon reasonable request.
